# Expression of urease by *Haemophilus influenzae *during human respiratory tract infection and role in survival in an acid environment

**DOI:** 10.1186/1471-2180-11-183

**Published:** 2011-08-16

**Authors:** Timothy F Murphy, Aimee L Brauer

**Affiliations:** 1 Division of Infectious Diseases, Department of Medicine, 701 Ellicott Street, University at Buffalo, State University of New York, Buffalo, NY 14203, USA; 2 Department of Microbiology, 701 Ellicott Street, University at Buffalo, State University of New York, Buffalo, NY 14203, USA; 3 New York State Center of Excellence in Bioinformatics and Life Sciences; 701 Ellicott Street, University at Buffalo, State University of New York, Buffalo, NY 14203, USA

## Abstract

**Background:**

Nontypeable *Haemophilus influenzae *is a common cause of otitis media in children and lower respiratory tract infection in adults with chronic obstructive pulmonary disease (COPD). Prior studies have shown that *H. influenzae *expresses abundant urease during growth in the middle ear of the chinchilla and in pooled human sputum, suggesting that expression of urease is important for colonization and infection in the hostile environments of the middle ear and in the airways in adults. Virtually nothing else is known about the urease of *H. influenzae*, which was characterized in the present study.

**Results:**

Analysis by reverse transcriptase PCR revealed that the *ure *gene cluster is expressed as a single transcript. Knockout mutants of a urease structural gene (*ureC*) and of the entire *ure *operon demonstrated no detectable urease activity indicating that this operon is the only one encoding an active urease. The *ure *operon is present in all strains tested, including clinical isolates from otitis media and COPD. Urease activity decreased as nitrogen availability increased. To test the hypothesis that urease is expressed during human infection, purified recombinant urease C was used in ELISA with pre acquisition and post infection serum from adults with COPD who experienced infections caused by *H. influenzae*. A total of 28% of patients developed new antibodies following infection indicating that *H. influenzae *expresses urease during airway infection. Bacterial viability assays performed at varying pH indicate that urease mediates survival of *H. influenzae *in an acid environment.

**Conclusions:**

The *H. influenzae *genome contains a single urease operon that mediates urease expression and that is present in all clinical isolates tested. Nitrogen availability is a determinant of urease expression. *H. influenzae *expresses urease during human respiratory tract infection and urease is a target of the human antibody response. Expression of urease enhances viability in an acid environment. Taken together, these observations suggest that urease is important for survival and replication of *H. influenzae *in the human respiratory tract.

## Background

Nontypeable (non encapsulated) *Haemophilus influenzae *is an exclusively human pathogen whose primary ecological niche is the human respiratory tract. *H. influenzae *is a common and important human pathogen, causing otitis media in children and lower respiratory tract infection in adults with chronic obstructive pulmonary disease (COPD) [1-3]. The course of COPD, the fourth leading cause of death in the world, is characterized by intermittent worsening called exacerbations. Approximately half of exacerbations are caused by bacterial infection, with *H. influenzae *being the most frequent bacterial cause [2]. In addition to causing exacerbations, *H. influenzae *also chronically colonizes the lower airways of adults with COPD. The normal human respiratory tract is sterile below the vocal cords, as determined by culture. However, in adults with COPD, the lower airways are colonized by bacteria, with *H. influenzae *as the most common pathogen in this setting [4-7].

The human respiratory tract is a hostile environment for bacteria. Nutrients and energy sources are limited. In the setting of COPD, airways are characterized by an oxidant/antioxidant imbalance and by an inflammatory milieu [8-12]. Thus to survive and cause infection in the human respiratory tract, *H. influenzae *must express proteins and other molecules to enable persistence in this unique environment.

In previous work, we characterized the proteome of *H. influenzae *that was grown in pooled human sputum obtained from adults with COPD in an effort to simulate the environment of the human airways in COPD [13]. In comparison to the same strain of *H. influenzae *grown in chemically defined media, 31 proteins were present in greater abundance in sputum grown-conditions at a ratio of > 1.5 compared to media-grown conditions. These included antioxidant proteins, stress response proteins, proteins that function in the uptake of divalent cations and proteins that function in the uptake of various molecules. Interestingly, the second most abundant protein with regard to the ratio of sputum-grown to media-grown analysis was urease C, the alpha subunit of urease, which was present in an abundance of 7-fold greater in sputum-grown conditions compared to media-grown conditions. This is an interesting finding in light of the observation by Mason et al [14] who monitored gene expression by *H. influenzae *in the middle ear of a chinchilla, the most widely used animal model of otitis media. The gene that encodes urease accessory protein, *ureH*, was induced 3.9-fold in bacterial cells in the middle ear compared to baseline. These two genes, *ureC *and *ureH *are part of the urease gene cluster and were among the most highly up regulated genes. These observations suggest that expression of urease is important for survival and growth of *H. influenzae *in the respiratory tract.

Ureases are nickel dependent enzymes that catalyze the hydrolysis of urea to form ammonia and carbon dioxide [15,16]. Urease is best studied as a virulence factor in *Helicobacter pylori *which colonizes the stomach and *Proteus mirabilis *which causes urinary tract infections [17-23]. Urease is also important for survival and pathogenesis of several bacterial species [24-27]. Urease functions to raise the pH of the environment, allowing survival in acidic media; urease also enables bacteria to use urea as a sole nitrogen source [28]. While these are the best known functions of urease, this protein also interacts with the human host and acts as virulence factor by several other mechanisms, including activation of macrophages [29], induction of inflammatory mediators [30-32], dysregulation of gastric epithelial tight junctions [33], apoptosis [34], activation of platelets, enhanced survival in macrophages [35,36] and others [37,38].

Virtually nothing is known about the urease of *H. influenzae*. In view of the high degree of up regulation of urease expression by *H. influenzae *in the respiratory tract and the importance of urease as a virulence factor in other bacteria, the goal of this study is to characterize the urease of *H. influenzae*. In particular we have constructed knockout mutants of *ureC *and the urease operon to assess urease activity by *H. influenzae*, characterized the urease transcript, determined the optimal pH for urease activity and demonstrated that the urease operon is present in clinical isolates from otitis media and COPD. Analysis of pre and post infection serum samples from adults with exacerbations of COPD caused by *H. influenzae *demonstrated directly that urease is expressed during human infection. Finally, we demonstrate that urease activity enhances survival of *H. influenzae *at a reduced pH.

## Results

### Identification of urease gene cluster

**Figure 1:**
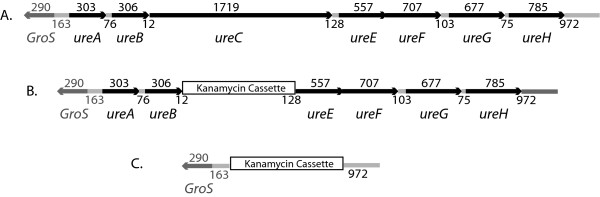
**1A. Diagram of urease gene cluster**. Numbers above genes indicate length of genes in nucleotides and numbers below indicate nucleotides between gene coding sequences. **1B**. Diagram of *ureC *knockout mutant. 1C. Diagram of urease operon knockout mutant.

The α subunit of urease, which was present in increased abundance in *H. influenzae *grown in pooled human sputum based on proteomic analysis, is a protein of 572 amino acids with a predicted molecular mass of 62 kilodaltons that is encoded by *ureC *[13]. The *ureC *gene is the third gene in the urease gene cluster, (Figure 1A); *ureA *and *ureB *encode the γ and β subunits respectively and *ureE, ureF, ureG *and *ureH *encode urease accessory proteins. These genes correspond to loci HI0535 through HI0541 in *H. influenzae *strain KW20 Rd (GenBank L42023.1) and to loci NTHI 0661 through NTHI 0667 in *H. influenzae *strain 86-028NP (GenBank CP000057).

### Characterization of mutants

**Figure 2:**
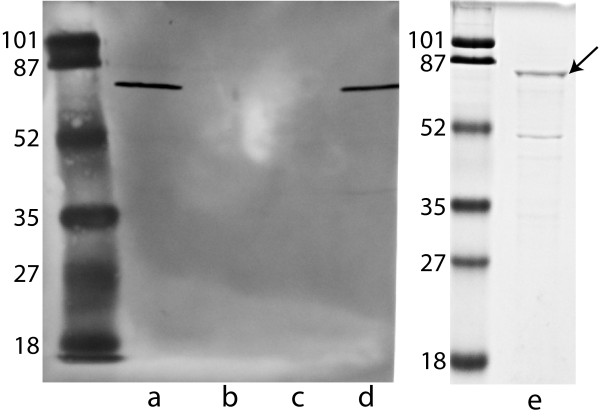
**Characterization of mutants and recombinant urease C protein**. Left panel. Immunoblot assay probed with rabbit antiserum (1:50,000) raised to recombinant purified urease C and adsorbed with urease mutant 11P6H*ureC*^-^. Blots were probed with goat anti-rabbit IgG (1:1000) and color was developed with horseradish peroxide developer. Lanes contain whole cell lysates as follows: a) Wild type 11P6H; b) Urease C mutant 11P6H*ureC*^-^; c) Urease operon mutant 11P6H*ure*^-^; d) Complemented urease C mutant 11P6H*ureC*^-^(p*ureC*). Right panel. Coomassie blue stained polyacrylamide gel. Lane e) Purified recombinant urease C. Arrow denotes full size protein. The lower band is a fragment of the full size protein. Molecular mass standards are noted on the left of each panel in kilodaltons.

A *ureC *mutant was constructed in our prototype COPD exacerbation strain 11P6H by replacing the *ureC *gene with a non polar kanamycin resistance cassette by homologous recombination using overlap extension PCR (Figure 1B). The mutant construct was confirmed by PCR using oligonucleotide primers in and around the gene in the wild type strain and the kanamycin cassette in the mutant, and by sequencing through the region of homologous recombination. An immunoblot assay of whole cell lysates probed with rabbit antiserum raised to recombinant urease C reveals the presence of an ~62 kDa protein band in the wild type strain, corresponding to urease C, and no bands in the *ureC *mutant (Figure 2).

Complementation of the *ureC *mutation was accomplished by cloning a fragment corresponding to the promoter region of the urease operon upstream of *ureA *through *ureC *into plasmid pSPEC and transforming the plasmid into the *ureC *mutant [39]. The complemented mutant expresses urease C detected by specific antiserum (Figure 2, lane d).

A knockout of the entire urease gene cluster was constructed using a similar overlap extension PCR strategy (Figure 1C). The mutant construct was confirmed by PCR and sequencing through the region of homologous recombination. An immunoblot assay of the whole bacterial cell lysate of the urease operon mutant probed with antiserum to urease C reveals an absence of a urease C band (Figure 2, lane c) that is present in wild type.

**Figure 3:**
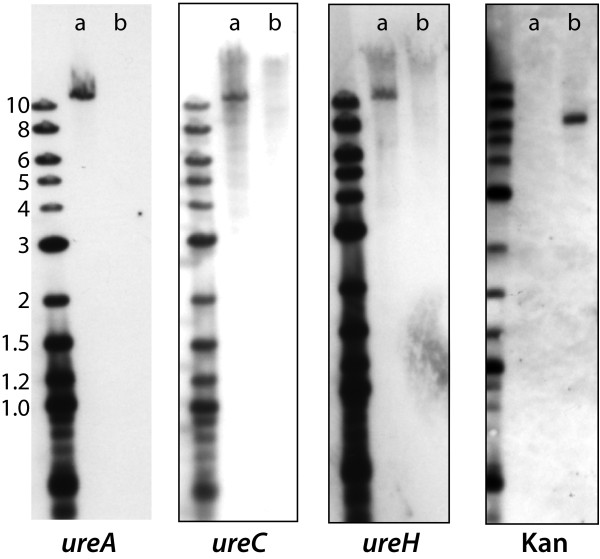
**Southern blot assay**. Purified genomic DNA of *H. influenzae *was restricted with *Eco*RI and hybridized with 200 bp probes corresponding to *ureA, ureC, ureH *and kanamycin cassette (kan) as noted at the bottom of each panel. Lanes a) wild type strain 11P6H; lanes b) urease operon mutant 11P6H*ure*^-^. Molecular size markers are noted on the left in kilobases.

To further characterize the urease operon mutant, genomic DNA from wild type and urease operon mutant strains was purified, restricted with *Eco*R1 and subjected to Southern blot assay. Probes that corresponded to the amino terminal region (*ureA*), the central region (*ureC*) and the carboxy terminal region (*ureH*) of the gene cluster and the kanamycin cassette revealed an absence of each of these 3 genes in the mutant and the presence of a kanamycin cassette as expected (Figure 3).

### Characterization of purified recombinant urease C

Recombinant urease C was purified by elution from a metal affinity column and refolded by sequential dialysis in buffers that contained decreasing concentrations of arginine. Analysis of the purified protein by SDS PAGE showed a prominent band at the predicted size (Figure 2, lane e). Preparations of the purified protein also revealed a second band of varying intensity of a lower molecular mass. Immunoblot assay with antibody that recognizes the 6 histidine tag detected both bands, indicating that the smaller band resulted from proteolytic degradation of the full length protein (data not shown). Protease inhibitors were used during purification and storage; however the purified protein was prone to proteolytic degradation. The purified recombinant protein was used to raise antiserum in rabbits and to measure antibody by ELISA in human serum. Thus, this level of proteolytic degradation would not be expected to adversely affect these experiments.

### Characterization of urease activity

**Figure 4:**
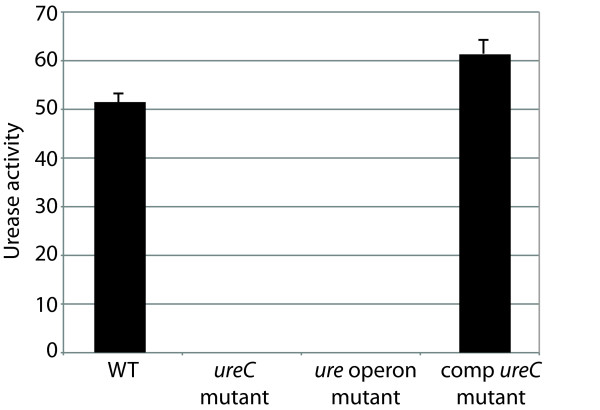
**Urease activity of mutants**. Results of urease assays with wild type, mutants and complemented mutant as noted at bottom. Urease activity is expressed on the Y-axis as μmoles of urea hydrolyzed per minute. Results are the mean of 3 independent assays and error bars denote standard deviation. Urease activity was undetectable in *ureC *mutant and *ure *operon mutant.

Crude cell extracts of *H. influenzae *11P6H were used to determine urease activity in wild type 11P6H and mutant strains. The *ureC *knockout mutant and the urease operon mutant both demonstrated no detectable urease activity compared to wild type and *ureC *complemented mutant when grown in laboratory media (Figure 4). We conclude that the *ureA-H *gene cluster accounts for all detectable urease activity of *H. influenzae *under the conditions of this assay. In addition, knocking out *ureC *alone, which encodes the major structural subunit of urease, completely abrogates urease activity.

**Figure 5:**
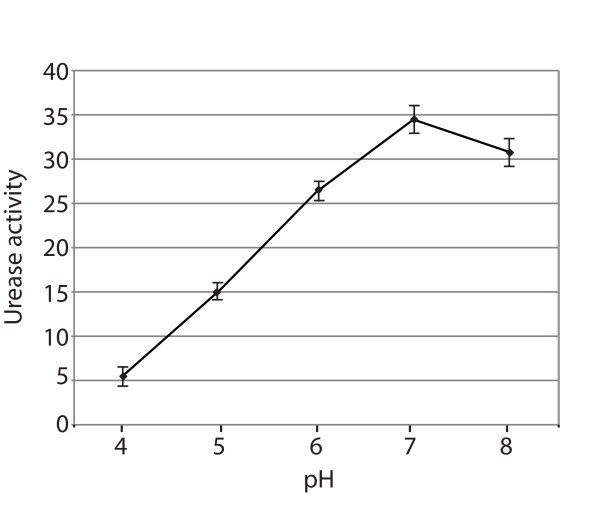
**Optimal pH of urease activity**. Urease activities of *H. influenzae *protein extracts were assayed in buffers of varying pH as noted on X-axis. Y-axis is urease activity in μmols of urea hydrolyzed per min. Each point is the average of 3 independent experiments and error bars indicate standard deviations.

The optimal pH of *H. influenzae *urease was determined by preparing whole cell extracts in phosphate buffers ranging in pH from 4 to 8. The optimal pH for urease was 7, with marked reduction in activity at lower pH (Figure 5).

**Figure 6:**
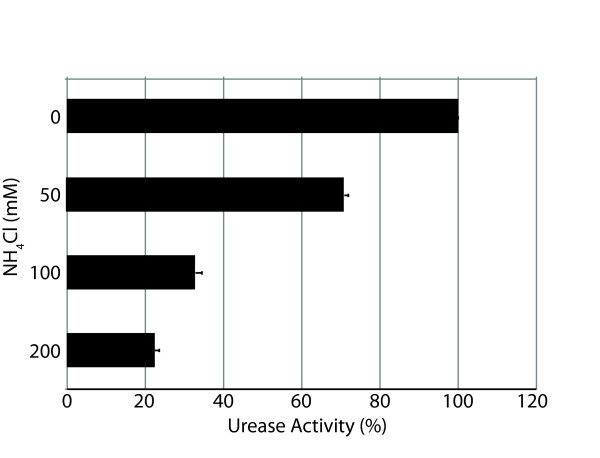
**Expression of urease**. Urease activity in the presence of varying concentrations of ammonium chloride as noted on the Y-axis. Results are expressed as a per cent of maximum activity (X-axis) in the absence of added ammonium chloride. Each bar is the average of three independent experiments and the error bars indicate standard deviations.

To begin to assess factors that control urease expression in *H. influenzae*, the effect of nitrogen availability on urease production was measured by adding increasing concentrations of ammonium chloride to bacteria growing in broth culture. Urease production decreased as the concentration of added ammonium chloride increased (Figure 6).

### Analysis of urease transcript

**Figure 7:**
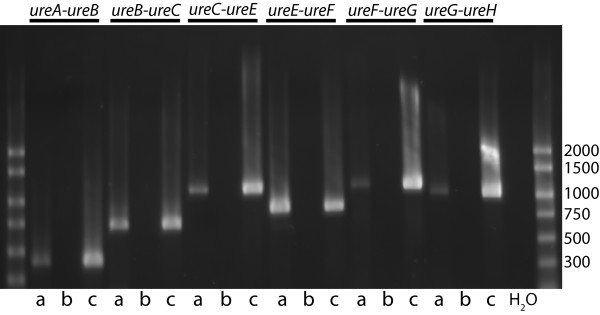
**Reverse transcriptase PCR of urease operon**. Ethidium bromide stained agarose gel showing results of reverse transcriptase PCR with *H. influenzae *strain 11P6H. Lanes: a) purified RNA with reverse transcriptase and *Taq *DNA polymerase; b) purified RNA with *Taq *DNA polymerase (negative control); c) purified DNA with *Taq *DNA polymerase (positive control). Lane H_2_O is a water control. Oligonucleotide primers were designed to span adjacent genes in the gene cluster as noted at the top of the gel (See Table 1). Molecular size markers are noted in base pairs on the right.

Reverse transcriptase PCR was performed to determine whether genes *ureA *through *ureH *of the urease gene cluster are transcribed as a single transcript or as multiple transcripts. Reverse transcriptase PCR was performed using RNA isolated from *H. influenzae *11P6H grown in broth using primers designed to correspond to transcripts that would span adjacent genes in the gene cluster (Figure 1). Figure 7 shows that the genes of the urease gene cluster are transcribed as a single transcript. Control assays confirmed that the purified RNA was free of contaminating DNA (Figure 7, lanes b).

### Presence of urease operon in clinical isolates

**Figure 8:**
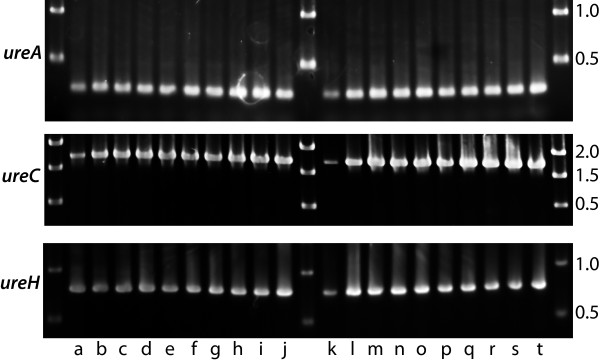
**Urease operon in clinical isolates**. Ethidium bromide stained agarose gels showing amplicons of genes in the urease gene cluster as noted on the left. Lanes a through j, amplicons from COPD sputum isolates 6P8H1, 14P14H1, 24P17H1, 27P5H1, 33P18H1, 43P2H1, 55P3H1, 66P33H1, 74P16H1, 91P18H1, respectively. Lanes k through t, middle ear fluid aspirate isolates 1749, 1826, 6699, 6700, 4R, 17R, 26R, 47R, P86, P113, respectively. Molecular size standards are noted on the right in kilobases.

To determine whether the urease operon is present in clinical isolates of *H influenzae*, 20 clinical isolates, including 10 otitis media strains and 10 COPD strains were studied by PCR. Primers corresponding to genes located in the 5' region (*ureA*), central region (*ureC*) and 3' region (*ureH*) of the operon were designed. Amplicons of identical size were obtained from 20 of 20 clinical isolates with all 3 sets of primers (Figure 8). These results indicate that the urease operon is present in all strains tested and that no variation was observed in the lengths of these genes in diverse strains tested.

A BLASTn search with the sequence corresponding to the urease operon was performed to determine which strains of *H. influenzae *whose genomes are available in GenBank contained the urease operon. Five of 6 strains whose complete genome has been sequenced contain the urease operon. A high degree of sequence similarity in the urease operon is present among the 5 strains. In strain R2866, which is urease negative, the urease operon is replaced by a single gene with homology to the gonococcal *mtrF *gene [40]. Sequence analysis of the same region of 9 additional urease negative strains revealed sequence that is very similar to that of strain R2866 [40].

### Transcription of the *ureC *during growth in pooled human sputum

To assess expression of urease in conditions that simulate conditions in the human respiratory tract in COPD, transcription of *ureC *was measured in *H. influenzae *that was grown in the presence of pooled human sputum from adults with COPD in comparison to growth in the absence of human sputum using quantitative real time PCR. Results of *ureC *were normalized with *gyrA*, a gene that is constitutively expressed [14]. Transcription of *ureC *in media plus sputum was 3.32 ± 0.066 (mean ± standard deviation) fold greater than transcription of *ureC *in media alone (1.0 ± 0.223). We conclude that transcription of *ureC *is up regulated when *H. influenzae *grows in media with added human sputum compared to growth in laboratory media alone.

### Human antibody responses

To determine whether urease was expressed by *H. influenzae *during infection of the human respiratory tract, 18 serum pairs from patients who experienced exacerbations due to *H. influenzae *were assayed for the development of antibody to purified recombinant urease following exacerbation.

**Figure 9:**
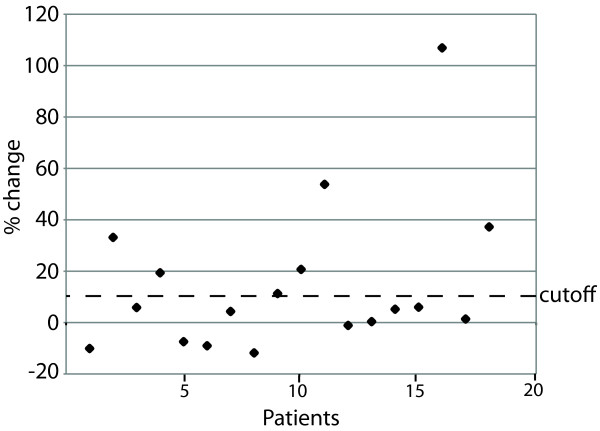
**Human antibody response to urease**. Results of ELISAs measuring serum IgG to purified recombinant urease C in serum samples from adults with COPD who experienced exacerbations due to *H. influenzae*. Patient numbers (N = 18) are noted on the X-axis. The per cent changes from pre exacerbation to post exacerbation are shown on the Y-axis. The cutoff value (dotted line) for a significant increase in antibody level was determined by averaging the difference between 8 control pairs of sera from patients who had negative sputum cultures and were clinically stable (see text).

The cutoff value for a significant percentage change between pre-exacerbation and post-exacerbation serum IgG levels was determined as previously described [41-44]. Eight control pairs of serum samples obtained 2 months apart (the same time interval for the experimental samples) from adults with COPD who were clinically stable and who had negative sputum cultures for *H. influenzae *were subjected to ELISA with the purified recombinant urease. The % change in OD_450 _values between the paired control samples was calculated. These paired control serum samples demonstrated a 3.36% ± 6.01 (mean ± SD) change when tested with urease. A change in OD of 9.37% represented the upper limit of the 99% confidence interval for the control samples. Therefore, any increase in value from pre to post exacerbation serum pairs of ≤ 9.37% was regarded as a significant change. A significant increase of serum IgG antibodies to urease was seen in 7 of 18 serum pairs (Figure 9). We conclude that *H. influenzae *expresses urease during infection of the human respiratory tract and is a target of human serum antibodies in adults with COPD.

### Susceptibility of *H. influenzae *to acid conditions

**Figure 10:**
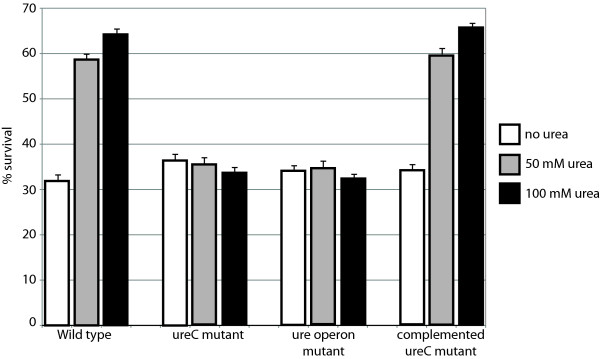
**Urease mediates survival at acid pH**. Survival of *H. influenzae *strain 11P6H and urease mutants at pH 4. Bacteria were suspended in buffer at pH 4 and incubated for 30 minutes at 37°C. Urea concentrations are as follows: white bars: no urea; gray bars: 50 mM urea; black bars: 100 mM. Bars indicate %survival calculated from colony counts performed at time 0 and 30 minutes. Values represent the mean of 3 independent experiments and error bars indicate standard deviation.

The ability of wild type and urease mutant to survive exposure to acid was investigated in the presence and absence of urea. Incubation of *H. influenzae *at pH 4 in the absence of urea, resulted in ~35% survival of wild type and mutant strains. However, in the presence of either 50 mM or 100 mM urea, survival of the wild type strain increased whereas no change in survival was observed in the urease C mutant or the urease operon mutant (Figure 10). Survival of the complemented mutant closely paralleled that of wild type, supporting the conclusion that urease mediates survival in acid conditions.

## Discussion

As an exclusively human pathogen, *H. influenzae *expresses molecules that mediate survival in the hostile conditions of the human respiratory tract. Previous studies in animal models and in conditions that simulate those in the human airways identified urease as a molecule that is expressed in high abundance by *H. influenzae*, providing evidence that urease plays a role in the pathogenesis of infection. Furthermore, urease activity may contribute to the pathogenesis of pulmonary infections due to *Actinobacillus pleuropneumoniae *in pigs [45]. These observations lead to the present study which is the first to characterize *H. influenzae *urease.

The *H. influenzae *urease gene cluster resembles that of other gram negative bacteria, possessing three contiguous structural genes (*ureA, ureB *and *ureC*) that encode the urease apoenzyme. Knocking out *ureC *alone by insertion of a nonpolar kanamycin cassette in its place resulted in complete loss of urease activity (Figure 4). Urease is a multi-subunit enzyme that requires an elaborate pathway for assembly in its active form. Associated with its three structural genes are 4 accessory genes which are necessary for synthesis of active enzyme. Based on available data from other organisms, *ureEFG *form a complex that keeps the apoenzyme in a conformation that will accept nickel. *H. influenzae ureH*, a structural homolog of ureD, is located downstream of the *ureEFG*, similar to the organization of the *H. pylori *urease gene cluster. *H. influenzae *does not have a *ureR *homolog, a regulatory gene that is present in some bacteria with urea-inducible urease [15]. Reverse transcriptase PCR demonstrated that the *H. influenzae *urease gene cluster is transcribed as a single transcript (Figure 7).

Urease activity in *H. influenzae *was dependent on nitrogen (ammonium chloride) availability as activity was maximal in the absence of added ammonium chloride and was markedly reduced as the concentration increased (Figure 6). This down regulation of urease expression by nitrogen sources is observed in other bacteria, including *Brucella abortus *and *Klebsiella aerogenes *and suggests that urease functions in assimilation of nitrogen from urea [23,25]. Because urea is translocated onto epithelial surfaces by secretory systems and in tissue exudates, urea is present in epithelial lining fluid of the human respiratory tract in concentrations approximately equal to that in plasma [46]. Thus, we speculate that the urease of *H. influenzae *facilitates nitrogen assimilation in the nutritionally limited environment of the human airways and the middle ear space.

Two indirect lines of evidence have suggested that *H. influenzae *expresses urease during human infection. Mason et al [14] showed that urease H is expressed during infection of the middle ear in chinchillas and Qu et al [13] showed that urease C was expressed in markedly increased abundance during growth in pooled human sputum. The present study advances those observations by showing directly that *H. influenzae *expresses urease during airway infection in adults who experienced exacerbations of COPD. Paired pre and post infection serum samples were subjected to ELISA with purified recombinant urease C to characterize the antibody response to urease following infection. Because the pre infection serum samples were collected one month prior to acquisition of the infecting strain of *H. influenzae*, an increase in the level of antibody to urease indicates the development of new antibodies following infection. All serum samples had detectable levels of antibody to urease and 7 of 18 patients developed significantly increased levels following infection compared to their own pre infection levels (Figure 9). This frequency of antibody response following bacterial infection is typical as heterogeneity in immune responses to bacterial antigens among individuals is a hallmark of COPD [47,48]. Note also that recombinant purified urease C was used in the ELISA and this protein is only one of 3 proteins that comprise the urease complex; thus, a urease C-based ELISA may underestimate the frequency of new antibody responses to urease following infection. These results indicate that *H. influenzae *expresses urease during exacerbations of COPD and that urease is a target of human antibody responses.

An important result from the present study is the observation that urease functions to mediate survival of *H. influenzae *in an acid environment. Urease mediates survival in low pH as a virulence mechanism in other bacteria, notably *H. pylori *which must survive in the stomach. Other selected respiratory pathogens express urease but the role of urease in pathogenesis of respiratory tract infection is unclear [49,50]. Microenvironments in the human respiratory tract are likely low pH, consistent with the speculation that the high level of expression of urease in the respiratory tract mediates survival in acid microenvironments. Furthermore, *H. influenzae *is now known to invade and persist in respiratory epithelial cells and macrophages, suggesting that withstanding lower pH in intracellular compartments may be a virulence mechanism [51-53].

## Conclusions

The present study demonstrates that 1) The *ureA-ureH *gene cluster of *H. influenzae *is exclusively responsible for urease production because knock out mutants show no urease activity. 2) Genes of the urease gene cluster are transcribed as a single transcript. 3) Urease expression is regulated in response to nitrogen availability. 4) The optimal pH for urease activity is 7.0. 5) The urease operon is present in all strains of *H. influenzae *tested including otitis media and COPD isolates. 6) Transcription of the *ure *operon is up regulated when *H. influenzae *grows in human sputum, consistent with the earlier observation established by proteomics analysis [13]. 7) Urease is expressed in the human airways during infection in adults with COPD and is the target of human antibody responses. And 8) Urease mediates survival of *H. influenzae *in an acid environment. In view of the high level of expression of urease in the respiratory tract, future work will focus on elucidating the role of urease as a virulence factor for *H. influenzae *infection of the human respiratory tract.

## Methods

### Bacterial strains and growth conditions

*H. influenzae *11P6H was isolated from the sputum of an adult with COPD who was experiencing an exacerbation as part of a prospective study at the Buffalo VA Medical Center [54]. The following strains were also isolated from the sputum of adults with COPD as part of the same study: 14P14H1, 24P17H1, 27P5H1, 33P18H1, 43P2H1, 55P3H1, 66P33H1, 74P16H1, 91P18H1. Each strain was isolated from a different subject.

*H. influenzae *strains 1749, 1826, 6699, 6700, 4R, 17R, 26R, 47R, P86 and P113 were isolated from middle ear fluid obtained by tympanocentesis from children with otitis media in either Buffalo NY or Rochester NY. All strains were identified as *H. influenzae *by growth requirement for hemin and nicotinamide adenine dinucleotide (NAD), absence of porphyrin production and absence of hemolysis. Each isolate was also subjected to immunoblot assay with monoclonal antibody 7F3 that recognizes outer membrane P6 to exclude the possibility of non hemolytic *H. haemolyticus *[55].

*H. influenzae *was grown on chocolate agar at 37°C in 5% CO_2 _or in brain heart infusion broth supplemented with hemin and NAD each at 10 μg/ml with shaking at 37°C. In selected experiments, *H. influenzae *was grown in chemically defined media (Table 1).

**Table 1:** Composition of chemically defined media (CDM)

Reagent	Concentration
NaCl	0.1 M
K_2_SO_4_	5.75 mM
Na_2_EDTA	4 mM
NH_4_Cl	4 mM
K2HPO_4_	2 mM
KH_2_PO_4_	2 mM
Thiamine HCl	6 μM
Thiamine pyrophosphate	1 μM
Pantothenic acid	8 μM
d-Biotin	12 μM
Glucose	0.5%
Hypoxanthine	0.375 mM
Uracil	0 .45 mM
L-aspartic acid	3.75 mM
L-glutamic acid HCl	7.5 mM
L-arginine	0.875 mM
Glycine HCl	0.225 mM
L-serine	0.475 mM
L-leucine	0.7 mM
L-isoleucine	0.225 mM
L-valine	0.525 mM
L-tyrosine	0.4 mM
L-cysteine HCl	0.35 mM
L-cystine	0.15 mM
L-proline	0.45 mM
L-tryptophan	0.4 mM
L-threonine	0.425 mM
L-phenylalanine	0.15 mM
L-asparagine	0.2 mM
L-glutamine	0.35 mM
L-histidine HCl	0.125 mM
L-methionine	0.1 mM
L-alanine	1.125 mM
L-lysine	0.35 mM
Glutathione reduced	0.15 mM
HEPES	42 mM
NaHCO_3_	0.125 mM
Na acetate trihydrate	6.25 mM
Choline chloride salt	0.05 mM
Myo-inositol	1 μM
MgCl_2_	2.5 mM
CaCl_2_	0.6 mM
Fe(NO_3_)_3_	0.1 mM
Nicotinamide adenine dinucleotide	0.02 mM
Protoporphyrin IX	0.02 mM
Histidine	6 μM
Triethanolamine	0 .01%

Chemically competent *E. coli *strains Top10 and BL21(DE3) were obtained from Invitrogen (Carlsbad, CA) and were grown at 37°C on Luria-Bertani (LB) plates or in LB broth supplemented with antibiotics as noted in individual experiments. Plasmid pSPEC1 was kindly provided by Lauren Bakaletz and Robert Munson [39].

### Construction of mutants

A mutant lacking *ureC*, the gene that encodes the alpha subunit of urease, was constructed using overlap extension PCR. The transforming DNA to accomplish this was composed of 3 fragments: 1) a ~1 kb fragment of DNA corresponding to sequence upstream of *ureC*, 2) the nonpolar kanamycin resistance cassette *Aph*A-3 [56], 3) a ~1 kb fragment of DNA corresponding to sequence downstream of *ureC*. Primers for each of the fragments were designed with 10 bp overlaps with complementary overlapping regions with the adjacent fragment (Table 2). The 3 fragments were amplified using the high fidelity DNA polymerase *Pfu *(Stratagene, Cedar Creek, TX) and were purified using the Qiaquick PCR purification kit (Qiagen, Valencia, CA). Amplicons were mixed in the absence of additional primers in a PCR with *Pfu *and were subjected to a PCR program consisting of 10 cycles with a denaturing step at 94°C for 30 sec, an annealing step at 50°C for 1 min and an elongation step at 72°C for 5 min. The fusion product was subsequently amplified by *Pfu *with primers 539frag1 5' and 539frag3 3' (Table 2). This amplicon consisted of 1020 bp sequence upstream of *ureC *and 1029 bp sequence downstream of *ureC *flanking the kanamycin cassette.

**Table 2:** Oligonucleotide primer sequences

Primer	Gene	Direction	Sequence^1^
**Construction of *ureC *mutant**			

539 frag1 5'	*ureC *upstream	Forward	5'-GACCTTTACCCACAGCTAAT-3'

539 frag1 3'	*ureC *upstream	Reverse	5'-TAGTTAGTCACTTGAAATTGTTA ATGCCAT-3'

539 frag2 5'	Kanamycin cassette	Forward	5'-CAATTTCAAGTGACTAACTAGGA GGAATAA-3'

539frag2 3'	Kanamycin cassette	Reverse	5'-TGACCCAATGCATTATTCCCTCC AGGTACT-3'

539 frag3 5'	*ureC *downstream	Forward	5'-GGGAATAATGCATTGGGTCAGC GATA-3'

539 frag3 3'	*ureC *downstream	Reverse	5'-ATCGCACACCGAGTTTG-3'

**Cloning of *ureC *into pSPEC1 to complement *ureC *mutation**			

539promoter F1	*ureA *upstream	Forward	5'-GAGAGGATCCGTAAAATTCGCTG ACTTTCG-3'

539C R1	*ureC *downstream	Reverse	5'-ATATGAATTCGCTACTTCACGCC CCGTA-3'

**Construction of urease operon mutant**			

539 Op frag1 F1	*ureA *upstream	Forward	5'-TACACCTTCCTTGCCCAC-3'

539 Op frag1 R1	*ureA *upstream	Reverse	5'-TAGTTAGTCAATTTTCATTCCTTAAT-3'

539 Op frag2 F1	Kanamycin cassette	Forward	5'-GAATGAAAATTGACTAACTAG GAGGAATAA-3'

539 Op frag2 R1	Kanamycin cassette	Reverse	5'-ACCAATTTTCCATTATTCCCTCCA GGTACT-3'

539Op frag3 F1	*ureA *downstream	Forward	5'-GGGAATAATGGAAAATTGGTAG GCTAT-3'

539Op frag3 R1	*ureA *downstream	Reverse	5'-CAGATGTTGCTTCAATTAAG-3'

**Subjecting multiple strains to PCR to assess presence of urease operon**			

UreaseA F1	*ureA*	Forward	5'-ATGCACTTAACTTCCAGAG-3'

UreaseA R1	*ureA*	Reverse	5'-TTATCTGATTGGATTATGC-3'

539 F1	*ureC*	Forward	5'-CAACATGGCATTAACAATTTCAAG-3'

539 R1	*ureC*	Reverse	5'-TTAGAATAGGAAATATCGCTG-3'

UreaseH F1	*ureH*	Forward	5'-ATGAACAGTAAATTATCC-3'

UreaseHR1	*ureH*	Reverse	5'-GAATTTGCTCTGCACGACA-3'

**Cloning of *ureC *gene to express recombinant protein**			

539 F1	*ureC*	Forward	5'-CAACATGGCATTAACAATTTCAAG-3'

539 R1	*ureC*	Reverse	5'-TTAGAATAGGAAATATCGCTG-3'

**Performing RT PCR**			

UreA-UreB F1	*ureA, ureB*	Forward	5'-ATGAGTGTAGCGGAAGTG-3'

UreA-UreB R1	*ureA, ureB*	Reverse	5'-ACGTTTAAACGCATTCCGC-3'

UreB-UreC F1	*ureB, ureC*	Forward	5'-TGAAACCAATAATGCCCT-3'

UreB-UreC R1	*ureB, ureC*	Reverse	5'-TTGCGTGCGTACCATCAGC-3'

UreC-UreE F1	*ureC, ureE*	Forward	5'-GGTATTGCGGAGCATATTGG-3'

UreC-UreE R1	*ureC, ureE*	Reverse	5'-GAATGACTGTGAGAATGCC-3'

UreE-UreF F1	*ureE, ureF*	Forward	5'-GCTTGAAACACGATGATG-3'

UreE-UreF R1	*ureE, ureF*	Reverse	5'-TGATTGCACCGACTGCTG-3'

UreF-UreG F1	*ureF, ureG*	Forward	5'-TGCCTTCGATGCAATGAAC-3'

UreF-UreG R1	*ureF, ureG*	Reverse	5'-AGCACCAACGAATGGAGC-3'

UreG-UreH F1	*ureG, ureH*	Forward	5'-AGTTTGCTTCCACCAGAGC-3'

UreG-UreH R1	*ureG, ureH*	Reverse	5'-GTTCGTCATTCAACACCC-3'

^1^underlined sequence denotes restriction enzyme site or cloning site

The fragment was transformed into *H. influenzae *strain 11P6H which was made competent by the method of Herriott et al [57] using the transformation protocol of Poje and Redfield [58] as previously described [59]. Transformants were selected on chocolate agar containing 15 μg/ml of robostimycin. A mutant was obtained (11P6H*ureC*^-^) and allelic exchange was verified by PCR analysis and sequencing as detailed in Results.

A mutant in which the entire urease gene cluster was knocked out and replaced with a kanamycin cassette was constructed (11P6H*ure*^-^) with the same strategy using the primers noted in Table 2. The mutant was verified by PCR analysis, Southern blot assay and sequencing.

### Complementation of *ureC *mutant

Complementation was accomplished by using the plasmid pSPEC1 [39]. A fragment containing the *ureC *gene and 740 bp upstream to include the promoter of the urease operon and 300 bp downstream was amplified from genomic DNA of strain 11P6H and ligated into pSPEC1 at *Bam*HI and *Eco*RI restriction sites (Table 2). After confirming the insert sequence of the resulting plasmid (p *UreC*spec), *H. influenzae *11P6H was electroporated with p *UreC*spec that had been methylated with CpG methylase (New England Biolabs) in a 0.1-cm cuvette (200 Ω, 2.5 kV, 25 μF). Cells were plated on chocolate agar containing 200 μg/ml of spectinomycin, incubated overnight and the complemented mutant 11P6H *ureC*^-^(p *UreC*spec) was obtained. This complemented mutant was grown in the presence of spectinomycin for all experiments.

### Southern blot assay

Southern blot assays were performed with genomic DNA restricted with *Eco*RI with the Hoefer TransVac vacuum blotting unit following the manufacturer's instructions (Hoefer, SanFrancisco, CA). Probes were biotinylated with the NEBlot Phototope kit (New England BioLabs) and blots were developed with the Phototope-Star Detection Kit (New England BioLabs) using the manufacturer's instructions.

### Measurement of urease activity

Urease activity was determined by measuring the amount of ammonia released from urea [25,60]. To prepare whole bacterial cell extracts, overnight cultures (5 ml) were centrifuged at 2500 × g for 10 min at 4°C and the pellet was suspended in 5 ml of phosphate buffered saline (PBS) pH 7.5. Cells were disrupted by sonication with three 10 second bursts (Branson Sonifier 450, output control 5). One ml of the resulting suspension was centrifuged at 16,000 × g for 2 min to remove unbroken cells and 10 μl of the sonic extract were added to 200 μl of PBS containing 50 mM urea and incubated at 37°C for 30 min. To perform the urease assay, 125 μl of sonic extract were mixed with 250 μl alkaline hypochlorite, 250 μl phenol nitroprusside and 1 ml of water and the assay was incubated for 30 min at 37°C. A volume of 200 μl was removed and placed into wells of a 96 well plate and the OD_595 _was measured in an ELISA plate reader. Urease activity was determined by the use of a standard curve using NH_4_Cl (0.156 mM to 2.5 mM) performed simultaneously with each assay. Urease activity was expressed in μmoles of urea hydrolyzed per minute.

### Expression and purification of recombinant protein encoded by *ureC*

The *ureC *gene was amplified by PCR from genomic DNA of *H. influenzae *strain 11P6H using oligonucleotide primers noted in Table 2 and cloned into pET101 D-TOPO (Invitrogen, Carlsbad, CA), which places a 6 histidine tag on the carboxy terminus of the recombinant protein, using manufacturer's instructions. Chemically competent *E. coli *TOP10 cells were transformed with the recombinant plasmid and transformants were selected by plating on LB plates containing 50 μg/ml of carbenicillin. The plasmid (p539) from a transformant was confirmed to have the *ureC *gene by PCR and by sequence determination. Plasmid p539 was purified using the Qiagen plasmid mini purification system and transformed into chemically competent *E. coli *BL21(DE3) for expression. To express recombinant protein, 2.5 ml of overnight culture was used to inoculate 50 ml of LB broth containing 300 μg/ml of carbenicillin. When the culture reached an OD_600 _of ~0.6, expression was induced by the addition of IPTG to a concentration of 4 mM. Cells were harvested by centrifugation after 4 hours and recombinant protein was purified with Talon Metal Affinity resin (Clontech, Mountain View, CA) using manufacturer's instructions.

The purified recombinant protein was refolded by dialysis in buffer with sequentially decreasing concentrations of L-arginine. The buffer contained 0.15 M NaCl, 20 mM tris pH 9, with decreasing concentrations (1 M, 0.5 M, 5 mM) of L-arginine. Protease Arrest™ (EMD Chemicals, Gibbstown NJ) was added to purified protein.

### Development of antiserum to urease C

Purified recombinant urease C was sent to Covance (Denver, PA) for antibody production in New Zealand white rabbits using a 59 day protocol. All applicable regulations for animal treatment were followed in accordance with the recommendations in the Guide for the Care and Use of Laboratory Animals of the National Institutes of Health (http://oacu.od.nih.gov/regs/guide/guide.pdf). Specific pathogen free rabbits received 250 μg of purified protein with complete Freund's adjuvant subcutaneously on day 0, followed by 125 μg of protein with incomplete Freund's adjuvant subcutaneously on days 21 and 49. Blood was obtained on day 59.

The rabbit antiserum was adsorbed with 11P6H *ureC*^- ^(urease C mutant) to remove background rabbit antibodies to *H. influenzae*. To accomplish this, bacteria were grown to log phase in broth, centrifuged to pellet bacteria, washed in PBS and suspended in 1 ml of a 1:1000 dilution of rabbit antiserum. After incubation for 30 min at 4°C, bacteria were removed by centrifugation. This process was repeated 3 more times. After the last adsorption, the serum was filter sterilized.

### Reverse transcriptase-PCR

Bacteria were grown in chemically defined media (Table 1) and RNA was isolated using a QIAGEN RNeasy kit and a Qiashredder column (QIAGEN, Valencia, CA) following the manufacturer's instructions, with an additional incubation with RNase-free DNaseI (Promega) for 30 min at 37°C. Reverse transcriptase PCR (RT-PCR) was performed using a QIAGEN OneStep RT-PCR kit and RNaseOut inhibitor (Invitrogen, Carlsbad, CA). Primers were designed to amplify fragments that would be predicted to correspond to transcripts that span adjacent genes in the urease gene cluster (Table 2). To exclude the possibility of contaminating DNA, parallel reactions with *Taq *DNA polymerase (HotMaster Mix; Eppendorf, Hamburg, Germany) were performed. Following amplification, samples were electrophoresed in 1% agarose gels and stained with ethidium bromide.

### COPD Study Clinic

The COPD study clinic at the Buffalo Veterans Affairs Medical Center is an ongoing prospective study that was started in 1994 [54]. The study was approved by the Health Sciences Institutional Review Board of the University at Buffalo and the Human Studies Subcommittee of the Western New York Veterans Affairs Healthcare System. All study participants provided written informed consent. To be included in this study, patients must have chronic bronchitis as defined by the American Thoracic Society [61] and must be willing to attend the study clinic monthly. Patients with asthma, malignancies, or other immunocompromising illnesses were excluded. Patients were seen monthly and at times when an exacerbation was suspected. At each visit clinical criteria were used to determine whether patients were experiencing an exacerbation or whether they were clinically stable as previously described [54]. Additionally at each visit, serum and expectorated sputum samples were collected. Bacteria present in the sputum were identified using standard techniques. Serum and bacteria obtained from sputum cultures were stored at -80°C.

An exacerbation of COPD caused by *H. influenzae *was defined by the onset of clinical symptoms of an exacerbation simultaneous with the acquisition of a new strain of *H. influenzae *that had not previously been isolated from that patient based on molecular typing [54]. Serum samples collected one month prior to acquisition of the strain and one month following the exacerbation were used to analyze human serum antibody responses to the purified recombinant urease C.

### Pooled human sputum

Expectorated sputum samples were collected from subjects in the COPD Study Clinic and were processed for culture as previously described [54,62]. Briefly, sputum samples were homogenized by incubation at 37°C for 15 minutes with an equal volume of 0.1% dithiothreitol. After an aliquot was removed for quantitative culture, sputum samples were centrifuged at 27,000 × g for 30 minutes at 4°C and supernatants were stored at -80°C until used. Samples from patients who were receiving antibiotics and samples that grew potential pulmonary bacterial pathogens in culture were excluded. Supernatants from approximately 100 sputum samples from 30 individuals were pooled for the purpose of growing bacteria in pooled sputum supernatants [13]. To render the sputum supernatants sterile, the pooled samples were placed in Petri dishes and exposed to UV light in a cell culture hood for approximately 10 minutes. An aliquot was plated on chocolate agar and no growth was detected after overnight incubation.

### Quantitative real time PCR

*H. influenzae *was grown in the presence pooled human sputum from adults with COPD to simulate conditions in the human respiratory tract. To assess transcription of *ureC*, strain 11P6H was grown overnight in chemically defined media (CDM) at 37°C with shaking to which pooled human sputum supernatant of 20% of the volume of the culture was added [13]. A second culture was grown simultaneously in CDM to which PBS containing 0.1% dithiothreitol was added to 20% of the total volume as a control for the sputum supernatant. Cells were harvested by centrifugation at 10,000 × g for 10 minutes at 4°C. Cells were washed by suspending in cold PBS and centrifuging again using the same conditions. Bacterial RNA was isolated as described above (Reverse Transcriptase-PCR).

Quantitative real time PCR was performed using the BioRad MyiQ Real-Time PCR Detection System. Oligonucleotide primers pairs (Table 2) were designed with Primer 3 software. Each reaction mixture contained 5 ng purified RNA, 100 nM of each primer, 12.5 μl 2 × Sybr Green Supermix (BioRad), 0.125 μl reverse transcriptase and 6.375 μl water. Controls lacking reverse transcriptase or RNA template contained the appropriate volume of water in place of enzyme or template. Each purified RNA sample was tested for DNA contamination prior to proceeding with the real time PCR assay. Results with *gyrA*, a constitutively expressed gene, were measured in both growth conditions and used to normalize the results with *ureC *in the corresponding growth condition. These normalized results were used to calculate the fold change expression of *ureC *during growth in CDM plus sputum compared to CDM alone. BioRad iQ5 software was used to analyze data.

### Enzyme-linked immunosorbent assay (ELISA)

Eighteen pre and post exacerbation serum pairs from adults with COPD followed in the COPD Study Clinic were subjected to ELISA to detect the development of new IgG antibodies in serum to urease C [48]. The change in antibody level from pre-exacerbation to post-exacerbation samples was calculated using the following formula: % change = [( post OD - pre OD )/pre OD] × 100. Paired pre-exacerbation and post-exacerbation samples were always tested in the same assay. The cutoff value for a significant percentage change between pre-exacerbation and post-exacerbation serum IgG levels was determined by studying 8 control pairs of serum samples obtained 2 months apart (the same time interval for the experimental samples) from patients who were clinically stable and who had negative sputum cultures for *H. influenzae *as described previously [42,43,48,63].

### Susceptibility of *H. influenzae *to acid

*H. influenzae *wild type and mutant strains were grown in broth to log phase, harvested by centrifugation and suspended to a concentration of ~10^7 ^colony forming units/ml in PBS adjusted to varying pH. Cells were incubated in the presence or absence of urea (50 mM or 100 mM) and dilutions of bacteria were plated at time 0 and at 30 min. Bacteria were counted after overnight incubation on chocolate agar.

## Authors' contributions

TFM was responsible for the conception and design of the study, analysis and interpretation of data, and drafting the manuscript. ALB made substantial contribution to the design of the study, acquired the data by performing the experiments and contributed important intellectual content to revisions of the manuscript. Both authors read and approved the final manuscript.
